# Covert Promotional Cancer-Related Content in Korean Search Engines: Computational Content Analysis of Naver and Google

**DOI:** 10.2196/90335

**Published:** 2026-09-29

**Authors:** Kyung Han You, Ho Young Yoon, Dalyong Kim, Joo Han Lim, Wonyoung Choi, Hyun Woo Lee, Sang-Cheol Lee

**Affiliations:** 1 Department of Media and Communication Studies, Jeonbuk National University, Baekjaedaero 567, Jeonju, Jeollabuk-do, Republic of Korea; 2 Division of Communication and Media, Ewha Womans University, Seoul, Seoul, Republic of Korea; 3 Dongguk University Ilsan Hospital, Goyang, Gyeonggi-do, Republic of Korea; 4 Inha University Hospital, Incheon, Incheon, Republic of Korea; 5 National Cancer Center, Goyang, Gyeonggi-do, Republic of Korea; 6 Ajou University Hospital, Suwon, Gyeonggi-do, Republic of Korea; 7 Soonchunhyang University Hospital Cheonan, 31 Suncheonhyang 6-gil, Dongnam-gu, Cheonan, Chungcheongnam-do, 31151, Republic of Korea, 82 1071093635

**Keywords:** digital cancer information, native advertising, covert promotion, health information quality, medical commercialization, digital health literacy, pinkwashing

## Abstract

**Background:**

Internet search engines serve as primary gateways to cancer information; yet, the commercialization of health content within organic search results remains understudied. While covert promotional content—such as native advertising and stealth marketing—has been documented in various contexts, systematic comparisons across structurally divergent search platforms are lacking.

**Objective:**

This study examined the prevalence, distribution, and information quality characteristics of covert promotional cancer-related content across Naver and Google, South Korea’s 2 dominant search engines, which have fundamentally different platform architectures.

**Methods:**

A 2-phase cross-sectional content analysis was conducted. Phase 1 used natural language processing to identify 34 cancer-related keywords from 1400 preliminary posts. Phase 2 systematically collected 5848 posts in October 2023, yielding 919 unique posts (598 from Naver and 321 from Google) that covered 7 major cancer types, collectively accounting for over 70% of Korean cancer incidence. Two trained coders analyzed promotional status, intensity, institutional sources, and information quality indicators (citation practices, information depth, and source attribution), with intercoder reliability exceeding κ=0.80. Chi-square tests were used to examine associations between platform and content characteristics across cancer type.

**Results:**

Covert promotional content appeared in 48.6% (447/919) of analyzed posts, with a significantly higher prevalence on Google (174/321, 54.2%) than on Naver (273/598, 45.7%; *χ*²_1_=5.78; *P*=.02). Platform differences were pronounced. Naver promotional posts predominantly originated from blogs (262/273, 96.0%) and exhibited full promotional intensity (126/242, 52.1%), while Google posts primarily came from hospital websites (141/174, 81.0%) with simple institutional identification (52/90, 57.8%). Institutional source distribution varied significantly by platform (*χ*²_5_=209.642; *P<*.001). Traditional medicine institutions dominated Naver (119/120, 99.2%), whereas university-affiliated hospitals predominated on Google (96/113, 85.0%). Information quality also differed substantially. Indirect citation was more common on Google (142/174, 81.6%) than on Naver (160/273, 58.6%; *χ*²_1_=25.653; *P<.*001), while comparative informational depth was higher on Google (97/174, 55.7%) versus Naver (53/273, 19.4%; *χ*²_2_=64.683; *P<.*001).

**Conclusions:**

Covert promotional cancer content is pervasive in Korean search results, with platform architecture systematically shaping promotional patterns, institutional sources, and information quality rather than reflecting deliberate marketing strategies. These findings underscore the need for platform-sensitive regulation and enhanced digital health literacy to protect vulnerable cancer information seekers from commercial exploitation embedded within ostensibly neutral search environments.

## Introduction

In contemporary health information environments, internet search engines have emerged as a primary gateway for patients, caregivers, and the general public to access medical knowledge. Particularly in contexts where formal health literacy may be limited, platforms such as Naver and Google are often the first point of contact for individuals seeking information on symptoms, treatment options, and health care providers. While the proliferation of health information online has democratized access to knowledge, it has also raised substantial concerns about the accuracy, reliability, and commercial neutrality of content found in search engine results.

These concerns are not confined to digital platforms alone but reflect a broader structural shift in contemporary health care communication. In an influential review, Schwartz and Woloshin [1] document the dramatic expansion of medical marketing in the United States between 1997 and 2016, as hospitals, pharmaceutical companies, and health care providers increasingly adopted marketing strategies to compete for patients and consumers. Importantly, this expansion was characterized not only by a rise in overt advertising but also by the growing use of informational and educational formats that blur the boundary between medical guidance and commercial persuasion. This commercialization of medical communication provides a critical backdrop for understanding why covert promotional content has become deeply embedded within ostensibly neutral health information environments, including internet search results.

The quality and accessibility of online health information are shaped not only by content producers but also by the platform infrastructures through which information is accessed. Search engines function as selective structures that systematically favor particular content formats, institutional sources, and informational styles through algorithmic ranking, user interface design, and business model integration [2,3], rather than through deliberate prioritization of commercial content; instead, these patterns may emerge from ranking criteria that favor institutional authority, content freshness, or user engagement metrics. These platform-level mechanisms create distinct information ecosystems that influence which health content becomes visible to users and is perceived as credible.

Recent scholarship in platform studies has emphasized that digital platforms are not neutral intermediaries but rather active infrastructures that shape communication practices and knowledge production [4-6]. In health communication contexts, this means that the same medical information may be presented, framed, and validated differently depending on the platform architecture. Understanding the structural selectivity is crucial for examining how medical commercialization manifests across different search environments, as platform design fundamentally shapes not only what information is visible but also how credibility and authority are signaled to users.

The South Korean digital health information landscape provides a theoretically meaningful context for examining platform-mediated medical commercialization. Unlike global search engines that primarily index external websites, Naver operates as a portal-based ecosystem deeply integrated with internally hosted user-generated content, including blogs, online communities (such as cafés), and question-and-answer forums [7]. This architectural difference incentivizes distinct forms of content production, where health care providers, alternative medicine practitioners, and commercial health actors actively maintain blog channels optimized for search visibility within Naver’s ecosystem.

Naver’s ranking algorithm emphasizes user engagement metrics—such as comments, likes, and visit frequency—and content freshness [8,9], which can systematically advantage frequently updated promotional health posts over static, evidence-based institutional sources. Additionally, the cultural salience of personal testimony and experiential knowledge in Korean health-seeking behavior aligns with Naver’s affordances for narrative-based content, potentially amplifying the visibility of promotional messages embedded in patient experience formats.

In contrast, Google search results have been described as favoring institutional websites, formal organizational presence, and signals associated with domain authority, through mechanisms such as PageRank and subsequent algorithmic updates [4,10]. This structural logic tends to surface hospital websites, medical institution pages, and established health organizations over user-generated blogs or community forums. However, institutional prominence does not guarantee transparency, as studies have documented that hospital websites frequently use sophisticated marketing strategies, including vague efficacy claims and selective information presentation [11].

Together, Google and Naver account for approximately 90% of search engine usage in South Korea. According to StatCounter (Statcounter Limited) [12], Google holds the largest overall market share in Korea, while data from Internet Trend (BizSpring Inc) [13] indicate that Naver maintains a higher share of active portal usage (57%), with Google accounting for approximately 33% of search traffic. This divergence reflects methodological differences between tracking platforms and underscores the complementary roles of the 2 search engines in Korean digital life. This duopolistic structure, combined with stark architectural contrasts between a portal-based ecosystem and a conventional search engine, makes Korea an ideal site for examining how platform infrastructure fundamentally shapes patterns of medical commercialization.

A growing concern is the increasing presence of covert promotional content, defined here as unpaid promotional posts from hospitals that are presented as informational content within organic search results and are distinct from paid native advertising. Unlike traditional advertising, which is explicitly labeled as such, covert promotional content takes the form of blog posts, patient testimonials, community forum discussions, or pseudojournalistic articles, thereby obscuring its commercial intent. This phenomenon—distinguished from native advertising or stealth marketing—has been particularly prevalent in cancer-related information, where emotional vulnerability and informational urgency heighten users’ susceptibility to misleading or biased content [14-16].

Recent research has documented the emergence of “patient influencers” in pharmaceutical marketing, in which authentic-looking testimonials about treatment experiences are used for promotional purposes without transparent disclosure of commercial relationships. Willis and Delbaere [17] characterize this as the “next frontier” in direct-to-consumer marketing, emphasizing how covert persuasion tactics embedded within seemingly independent patient narratives complicate users’ ability to distinguish commercial messaging from genuine health information sharing. This dynamic is amplified in cancer contexts, where patients’ intensive information-seeking behavior and decision-making urgency heighten their vulnerability to promotional content masquerading as educational resources.

Despite growing recognition of commercial influences in online health information, existing research has predominantly focused on content characteristics and marketing strategies, treating digital platforms as neutral distribution channels. This approach overlooks how platform architectures themselves systematically shape which forms of medical commercialization emerge, persist, and gain visibility. Previous studies have documented the prevalence of promotional health content [18,19] and analyzed disclosure practices in native advertising [20,21], but have rarely examined how platform-specific infrastructures produce distinct patterns of commercialization.

In the Korean context, where structurally divergent search platforms coexist, systematic comparison of commercialization across Naver and Google remains absent. Comparative analyses of search results between Korea and the United States have identified platform-level differences in health information reliability [7], yet the mechanisms through which platform architecture shapes promotional content distribution, institutional sources, and information quality have not been empirically examined. Moreover, whether platform differences in medical commercialization reflect strategic marketing adaptation by health care providers or structural selectivity embedded in search engine ecosystems remains an unresolved question with significant theoretical and policy implications.

In this regard, the present study addresses these gaps by conducting a systematic, platform-comparative content analysis of cancer-related search results from Naver and Google, focusing on 7 major cancer types that account for over 70% of cancer morbidity and mortality in South Korea, including colorectal, gastric, breast, prostate, liver, lung, and pancreatic cancers [22].

The proliferation of promotional health content online must be understood within the broader context of the commercialization of medicine. Over the past several decades, health care systems have increasingly adopted market-based logics, positioning patients as consumers [1,17,23-25] and medical services as competitive products, a shift theorized as the biomedicalization of health and illness [26]. This transformation has been extensively documented in studies of direct-to-consumer advertising, hospital branding, and the expansion of health care institutions’ marketing expenditures [1,23,24].

Scholars have argued that the commercialization of medicine extends beyond explicit advertising to encompass subtler forms of communicative influence, including educational materials, informational campaigns, and patient-oriented content that implicitly promote specific providers or treatments [1,17]. In this regard, medical communication has become a key site of marketization, where informational authority and commercial persuasion are increasingly intertwined [1,17].

A growing body of research has examined native advertising as a form of covert persuasion that mimics the appearance and tone of noncommercial content [14,15,20,21]. Unlike traditional advertisements, native ads are designed to blend into their surrounding informational environments, thereby reducing users’ awareness of persuasive intent and increasing message effectiveness [14,15]. Empirical studies have consistently shown that users frequently fail to recognize native advertising, particularly when disclosures are subtle, ambiguously worded, or visually inconspicuous [14,20,21,27].

In health communication contexts, native advertising raises distinct ethical concerns. Health-related native ads often adopt educational or testimonial formats, presenting themselves as neutral guidance while advancing commercial interests [17,23]. Prior studies have found that such content can influence treatment preferences, distort risk perceptions, and undermine informed decision-making, especially among users with limited health literacy [17,23].

Within search engine environments, covert promotional content often takes the form of blog posts, institutional web pages, or quasi-journalistic articles that provide general medical information before transitioning into institutional promotion [11,14,15,17]. These hybrid formats are particularly difficult for users to classify, as they occupy an ambiguous space between information and advertising [14,15,20].

Cancer represents a category of illness that is not only life-threatening but also deeply anxiety-inducing, prompting individuals to engage in intensive online information-seeking behavior. This behavioral tendency renders cancer-related content especially susceptible to commercialization. Health care institutions, including hospitals, alternative medicine clinics, and wellness centers, have increasingly leveraged online platforms to disseminate promotional messages under the guise of educational or informational content [23,24]. While such strategies may be legally permissible, their ethical implications remain contentious, particularly when the distinction between verified clinical guidance and commercial messaging becomes blurred.

Concerns about the commercialization of health information intersect closely with longstanding debates on information quality. Previous frameworks for evaluating online medical information have emphasized criteria such as accuracy, completeness, transparency of sources, and balance [16,18,19,28,29]. Subsequent research has demonstrated that online health information—particularly commercially affiliated content—often falls short of these standards, exhibiting vague or indirect citation practices, selective presentation of benefits, and a lack of clear source attribution [18,19].

In cancer-related contexts, where clinical complexity and emotional salience are high, such deficiencies may be especially consequential [19,23]. Recent studies further suggest that information quality cannot be assessed independently of platform context, as algorithmic ranking mechanisms and content affordances shape not only which information is visible but also how credibility is signaled [3-5]. As a result, information quality deficits may manifest differently across platforms, producing distinct configurations of epistemic risk rather than a simple dichotomy between “high-quality” and “low-quality” information.

To conceptualize covert promotional content, scholars have proposed typologies that categorize how commercial interests are obscured through moral, social, or informational framing [30-32]. Within health and illness contexts, one influential strand of research has examined “pinkwashing,” which refers to practices in which corporations or institutions align themselves with socially valued causes—such as cancer awareness or survivorship—while simultaneously advancing commercial objectives, particularly in relation to breast cancer awareness campaigns [33].

Although pinkwashing originated in analyses of cause-related marketing, its underlying logic is applicable to a broader range of covert promotional practices [30,31]. By framing commercial messages within narratives of care, hope, or public service, health-related promotional content can acquire moral legitimacy while evading scrutiny [30,31,33].

Taken together, previous research highlights the need to examine covert promotional health content as a structurally mediated phenomenon shaped by platform architectures, market logics, and information quality norms [1,3-5,14,18]. However, few empirical studies have systematically compared how these dynamics operate across different search engine ecosystems, particularly in national contexts characterized by strong domestic portals [7,11].

This study makes 3 original contributions to the literature. First, it provides a systematic, platform-comparative content analysis of covertly promotional cancer-related content across structurally divergent search engines operating within a single national context. Second, it identifies platform-associated differences in promotional patterns, institutional sources, and information quality that are consistent with a structural account of medical commercialization online. Third, it offers empirically grounded, platform-specific policy recommendations for regulating online medical advertising in domestic search engine ecosystems.

By analyzing cancer-related search results from Naver and Google, this study addresses the gap and advances our understanding of how platform-specific structures influence the commercialization and epistemic quality of online medical information. Drawing on prior research on the commercialization of health information and platform-mediated health communication, this study addresses the following research questions (RQs):

- RQ1. To what extent does covert promotional content appear in cancer-related search results on major search engines, and how does its prevalence differ across platforms?
- RQ2. How does the distribution of covert promotional content vary by cancer type across search engines?
- RQ3. How do institutional sources of covert promotional cancer-related content differ across search engines, and what structural patterns of medical commercialization emerge from these differences?
- RQ4. How do covert promotional cancer-related content items differ across search engines in terms of information quality characteristics, including citation practices, information depth, and source attribution?

## Methods

### Study Design

This study employed a cross-sectional quantitative content analysis to examine promotional cancer-related content in online search results using a 2-phase sequential design. Phase 1 used natural language processing (NLP) to identify keywords, while Phase 2 conducted systematic data collection. The final dataset comprised 919 content items from South Korea’s 2 dominant search platforms—Naver (57% market share) and Google (33% market share) [13]—covering 7 major cancers representing over 70% of Korean cancer incidence, including colorectal, gastric, breast, prostate, liver, lung, and pancreatic cancer [34]. For the purposes of this study, the term “content item” refers to any discrete unit of web-based content retrieved through search engine results, encompassing blog posts, hospital website pages, online community forum entries, news articles, and other publicly accessible web content formats. Where prior literature uses “posts” to refer to all such content types, this study adopts the more general term “content items” to reflect the heterogeneous nature of retrieved materials.

This descriptive and comparative content analysis addressed 4 RQs: (RQ1) the prevalence of covert promotional content in top-ranked cancer search results; (RQ2-RQ3) the associations of platform, cancer type, and institutional source with the distribution and intensity of covert promotional content; and (RQ4) information quality characteristics (citation practices, efficacy verification, information depth, and visual attribution) across search engines.

### Data Collection Procedure

#### Phase 1: Keyword Identification (September 7-16, 2023)

Preliminary data collection used API-based web scraping using Python (version 3.9; Python Software Foundation) to retrieve approximately 1400 posts (200 per cancer type) from exploratory searches. All search queries in both phases were conducted exclusively in Korean, using standard medical terminology (eg, colorectal cancer and breast cancer) that reflects real-world patient health information-seeking behavior. Searches retrieved the top 10 pages from Naver and the first screen from Google during business hours (9 AM-6 PM Korea Standard Time) using private browsing mode to eliminate personalization effects.

Korean text preprocessing and morphological analysis were conducted using *KoNLPy*’s Kkma analyzer [35], using a sequential processing approach: (1) noun extraction to isolate content-bearing terms, (2) stop word removal using a customized list of function words, particles, and domain-irrelevant terms, (3) word frequency analysis using term frequency counts across all retrieved posts per cancer type, and (4) co-occurrence pattern analysis to identify terms appearing across multiple cancer types. Terms appearing in the top 20 frequency ranks across all 7 cancer types were designated as universal keywords; cancer-specific keywords were identified based on high frequency ranks unique to each cancer type. This systematic NLP analysis identified 13 universal Korean keywords that appeared across all cancer types (early stage, health, cause, symptoms, screening/examination, immunity, treatment, surgery, metastasis, hospital, food/diet, management, and method) and 3 cancer-specific keywords per type, yielding 34 unique keywords that represent lay terminology. The complete keyword list, along with English translations, is presented in Table 1. This NLP-based approach is grounded in prior research demonstrating that frequently occurring terms in search engine results are systematically associated with dominant query patterns among users [36,37], as search engine algorithms rank content partially based on query-term alignment. We acknowledge that this represents a methodological inference rather than direct measurement of user queries and that future research could triangulate this approach with query log data where available.

**Table 1.** Top 20 most frequent keywords by cancer type identified through NLP^a^ analysis^b^.

Rank	Colorectal cancer	Breast cancer	Gastric cancer	Prostate cancer	Liver cancer	Lung cancer	Pancreatic cancer
1	Symptoms (63)^c^	Surgery (55)	Occurrence (54)	Treatment (50)	Treatment (62)	Treatment (52)	Symptoms (61)
2	Surgery (48)	Treatment (49)	Symptoms (49)	Test/screening (44)	Symptoms (36)	Hospital (45)	Early stage (48)
3	Hospital (47)	Hospital (48)	Surgery (47)	Male (44)	Hospital (33)	Symptoms (42)	Treatment (34)
4	Endoscopy (43)	Female (32)	Early stage (45)	Symptoms (40)	Surgery (29)	Survival (38)	Pain (31)
5	Treatment (42)	Insurance (29)	Treatment (37)	Occurrence (40)	Management (21)	Occurrence (33)	Hospital (30)
6	Occurrence (38)	Diagnosis (23)	Hospital (31)	Hospital (33)	Health (20)	Chemotherapy (31)	Surgery (28)
7	Early stage (36)	Symptoms (23)	Food/diet (29)	Survival (23)	Patient (20)	Surgery (30)	Health (27)
8	Patient (26)	Health (23)	Survival (28)	PSA^d^ (21)	Function (19)	Patient (29)	Occurrence (24)
9	Health (24)	Metastasis (22)	Good (26)	Surgery (21)	Occurrence (19)	Early stage (27)	Detection (24)
10	Receive treatment (24)	Management (21)	Endoscopy (25)	Good (19)	Receive treatment (19)	Diagnosis (27)	Cause (24)
11	Examination (24)	Examination (21)	Management (25)	Metastasis (19)	Liver disease (19)	Health (26)	Location (22)
12	Detection (23)	Early stage (20)	Receive treatment (24)	Method (17)	Benign (18)	Detection (24)	Survival (21)
13	Food/diet (22)	Receive treatment (20)	Metastasis (24)	Health (17)	Immunity (18)	Management (23)	Case (20)
14	Management (21)	Detection (19)	Intestinal metaplasia (24)	Prevention (16)	Early stage (18)	Immunity (22)	Diagnosis (20)
15	Good (20)	Occurrence (19)	Health (22)	Diagnosis (16)	Progression (17)	Smoking (21)	Patient (17)
16	Benign (19)	Patient (17)	High (21)	PSA level (15)	Risk (17)	Family (21)	Examination (17)
17	Accompanied by (19)	Screening (17)	Risk (20)	Receive treatment (15)	Disease (16)	Receive treatment (21)	Pancreas (17)
18	Method (19)	Method (17)	Case (17)	Information (15)	Examination (16)	Cause (20)	Good (17)
19	Case (19)	Survival (16)	Patient (16)	Compared to (14)	Information (15)	Examination (18)	Pancreatitis (16)
20	Compared to (18)	Case (16)	Many (16)	Tumor (14)	Absent (15)	Good (18)	Develop (16)

a NLP: natural language processing.

b Preliminary posts (n=1400; 200 per cancer type) retrieved from Naver and Google, South Korea, September 2023 (Phase 1).

c Numbers in parentheses indicate term frequency counts.

d PSA: prostate-specific antigen.

Based on the NLP frequency analysis results presented in Table 1, keywords were selected for Phase 2 data collection using 2 criteria. First, terms appearing among the top 20 frequency ranks across all 7 cancer types were designated as universal keywords (n=13): early stage, health, cause, symptom, screening/examination, immunity, treatment, surgery, metastasis, hospital, food/diet, management, and method. Second, terms with high-frequency ranks specific to each cancer type were designated as cancer-specific keywords (n=3 per type). Universal and cancer-specific keywords were combined with each cancer type name to generate the final set of Phase 2 search queries. Table 2 presents the complete list of final search queries used in Phase 2.

**Table 2.** Final phase 2 search queries by cancer type. All queries conducted in Korean; English translations provided (South Korea, October 2023).

Cancer type	Universal keywords (n=13)^a^	Cancer-specific keywords^b^
Colorectal cancer	Early stage, health, cause, symptom, screening/examination, immunity, treatment, surgery, metastasis, hospital, food/diet, management, and method	Endoscopy, polyp, and chemotherapy
Gastric cancer	Early stage, health, cause, symptom, screening/examination, immunity, treatment, surgery, metastasis, hospital, food/diet, management, and method	Endoscopy, intestinal metaplasia, and gastritis
Breast cancer	Early stage, health, cause, symptom, screening/examination, immunity, treatment, surgery, metastasis, hospital, food/diet, management, and method	Insurance, information, and cancer treatment
Prostate cancer	Early stage, health, cause, symptom, screening/examination, immunity, treatment, surgery, metastasis, hospital, food/diet, management, and method	PSA^c^, level, and tumor
Liver cancer	Early stage, health, cause, symptom, screening/examination, immunity, treatment, surgery, metastasis, hospital, food/diet, management, and method	Hepatitis, function, and progression
Lung cancer	Early stage, health, cause, symptom, screening/examination, immunity, treatment, surgery, metastasis, hospital, food/diet, management, and method	Cell, smoking, and family
Pancreatic cancer	Early stage, health, cause, symptom, screening/examination, immunity, treatment, surgery, metastasis, hospital, food/diet, management, and method	Pain, pancreatitis, and location

a Universal keywords (n=13) applied to all cancer types.

b Cancer-specific keywords (n=3 per type).

c PSA: prostate-specific antigen.

#### Phase 2: Systematic Data Collection (October 13-20, 2023)

Following a 3-week interval for NLP processing and protocol refinement, systematic searches combined each cancer type with identified keywords, generating 16 queries per cancer type (224 total queries across both platforms). For Naver, the top 10 result pages were retrieved per query, with each page displaying approximately 10 organic links, yielding up to 100 results per query. For Google, results displayed on the first screen (approximately 8‐10 organic links per screen) were collected. On average, 26.1 content items were retrieved per query (range: 15‐42), for a total of 5848 prior to deduplication. API-based scraping, conducted during weekday business hours, used standardized protocols and private browsing with a cleared cache. The collection excluded marked paid advertisements, focusing on organic results, including blogs (45%), hospital websites (25%), news articles (15%), forums (10%), and other formats (5%).

### Sample Processing

Automated URL-based deduplication reduced the number of posts from 5848 to 1159 unique posts. Eligibility screening applied inclusion criteria (Korean language, substantive cancer-related content, public accessibility, and identifiable digital format) and excluded posts requiring login (n=87), deleted/inaccessible content (n=109), and text-absent posts (n=44), yielding a final analytical sample of 919 posts. The platform distribution was Naver (598/919, 65.1%) and Google (321/919, 34.9%), with cancer type distribution ranging from 108 to 151 posts per type (11.8%‐16.4% each). Figure 1 presents the complete data collection flowchart.

**Figure 1.**
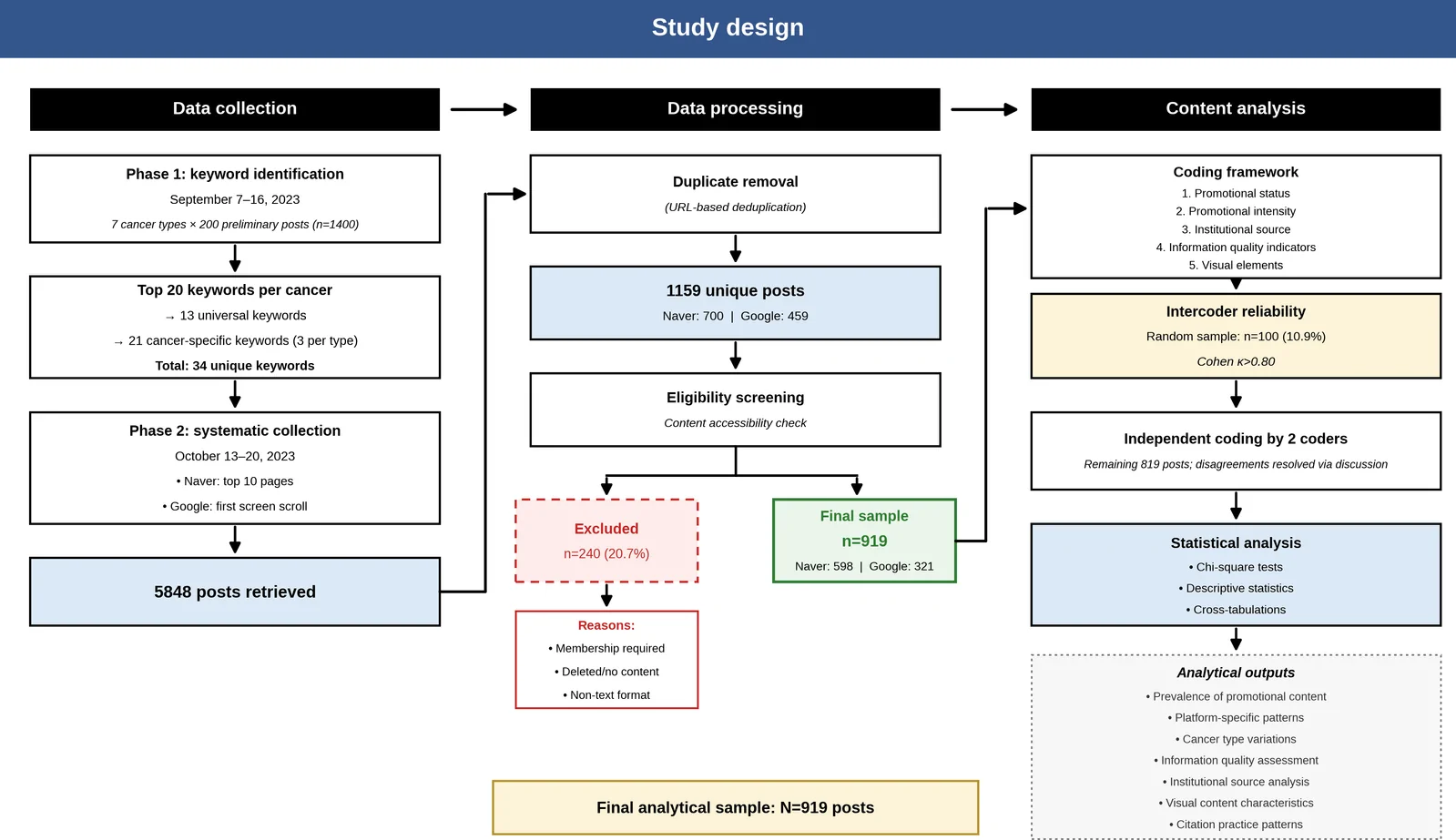
Two-phase data collection flowchart. Cross-sectional content analysis of covert promotional cancer-related content items on Naver and Google, South Korea, September-October 2023. Phase 1 (September 7‐16, 2023): natural language processing (NLP)–based keyword identification from 1400 preliminary posts. Phase 2 (October 13‐20, 2023): systematic data collection yielding 5848 retrieved items, deduplicated and screened to a final analytical sample of 919 content items (Naver n=598 and Google n=321).

### Operational Definition of Covert Promotional Content

Covert promotional content was operationally defined as posts containing hospital promotional intent presented as informational content or patient testimonials without transparent disclosure, consistent with previous studies [32]. Unlike transparently labeled paid advertisements, which are excluded from this analysis (Naver Power Links and Google Ads), covert promotional content appeared in organic search results without platform payment or advertising disclosure. This phenomenon parallels product placement in television programming or undisclosed sponsorship in social media, where commercial messages are embedded within content that appears editorially independent.

### Classification Criteria

Posts were classified as covert promotional content if any portion of the content served promotional purposes for the hospital, distinguishing them from purely informational posts. Covert promotional content included hospital website content promoting services, hospital-operated blogs describing treatments, and community posts presenting patient experiences while promoting specific institutions. Nonpromotional posts included patient support community testimonials without institutional promotion, educational content from medical professionals, and news articles featuring physician interviews about general treatment approaches.

### Types of Covert Promotional Content

Systematic coding identified 6 types of covert promotional content based on format and promotional intensity (see Figure 2):

1. Blog posts dominated by hospital promotion, where promotional content constituted the primary message.
2. Blog posts with partial hospital promotion, where promotional content comprised a secondary but identifiable component.
3. Community forum posts with overt promotional focus, where advertising objectives were explicit though not labeled as advertisements.
4. Patient experience narratives functioning as stealth marketing, where authentic-appearing testimonials about cancer treatment experiences served promotional purposes for specific hospitals. This type represented the most covert form, disguising commercial intent within seemingly genuine patient sharing.
5. Hospital website promotional content, where institutional sites presented treatment information with clear promotional elements.
6. Advertorial-format posts combining health information with promotional messages, where editorial-style cancer information incorporated promotional content at the introduction or conclusion.

**Figure 2.**
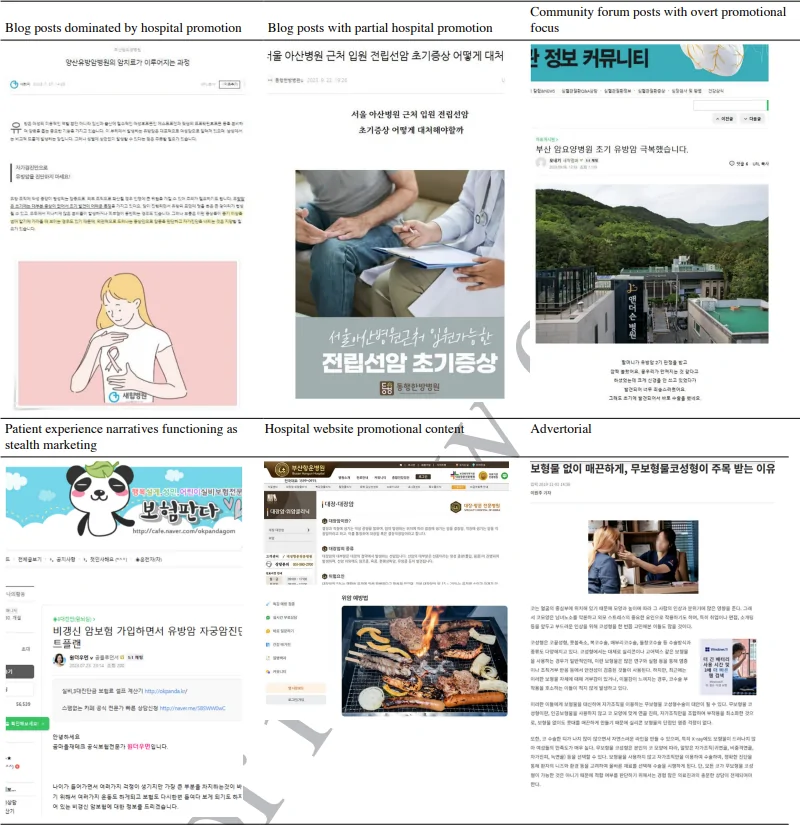
Type of covert promotional content.

### Level of Promotional Intensity

Among posts classified as covert promotional content, intensity was coded at 3 levels based on the proportion and prominence of promotional elements.

1. Simple identification: posts where the primary content is educational or informational in nature, with hospital affiliation indicated only through contact information (hospital name, phone number, address, and website) typically appearing at the header or footer. The hospital identification serves primarily as source attribution rather than promotional messaging.
2. Partial promotion: posts where promotional content comprises less than 50% of the total text, as determined by character count. These posts provide substantive health information while incorporating identifiable promotional elements such as treatment advantages, institutional expertise, facility descriptions, or patient testimonials that serve marketing purposes. Promotional and informational content are distinguishable but integrated within the same post.
3. Full promotion: posts where promotional content comprises 50% or more of the total text, as determined by character count. The primary focus is institutional promotion, treatment marketing, or facility showcasing, with health information serving primarily as a framing device for promotional messaging. These posts emphasize institutional branding, competitive advantages, or service promotion over neutral health education.

For partial and full promotion categories, promotional proportion was determined through systematic character counting using Python (version 3.9), measuring the ratio of promotional text (treatment marketing claims, institutional advantages, facility descriptions, testimonials with promotional framing) to total textual content.

### Temporal Design Rationale

The 6-week timeframe, spanning September to October 2023, was selected to allow adequate NLP processing time while minimizing seasonal variation. The 3-week interphase interval allowed for keyword extraction, validation, and protocol review. Weekday-only collection was used to standardize the timing of search collection, while fall timing avoided major Korean holidays (Chuseok) and year-end health campaigns that could introduce variation in promotional content.

### Coding Procedure of Content Analysis

#### Codebook and Training

A comprehensive codebook integrated deductive categories from health communication literature with inductive categories from a preliminary review of 50 posts, refined through 3 pilot testing iterations. Two native Korean-speaking graduate students in health communication underwent 8 hours of structured training, consisting of codebook review (2 hours), practice coding with discussion (3 hours), and expert-comparison coding (3 hours).

#### Reliability and Execution

Intercoder reliability was assessed using 100 randomly selected posts (10.9% of the sample) stratified by platform and cancer type. All variables had Cohen kappa (κ) values greater than 0.80, indicating excellent agreement [38]. After establishing reliability, the remaining 819 posts were randomly assigned to the coders. Weekly team meetings addressed ambiguous cases, with a third expert coder consulted to resolve disagreements.

#### Coding Dimensions

Five primary dimensions were coded: (1) promotional status (binary: promotional/nonpromotional); (2) promotional intensity (3-level: simple identification, partial promotion, and full promotion); (3) institutional source (6 categories: university-affiliated hospitals, small and medium-sized private hospitals, traditional Korean medicine long-term care hospitals, health policy and medical administration experts, nonexperts, and other); (4) information quality indicators (citation practices, efficacy verification, and information depth); and (5) visual elements (presence and source attribution).

### Sample Size

Sample size determination was guided by 3 considerations appropriate to content analysis research. First, pilot analyses indicated thematic saturation at 80‐100 posts per cancer type, with frequency distributions stabilizing for promotional strategies, institutional sources, and patterns of information quality. Second, the sample ensured adequate expected cell frequencies (>5) for chi-square tests across key comparisons, including platform (598 vs 321 posts), cancer type (7 categories with 108‐151 posts each), institutional source (6 categories), and promotional status (447 vs 472 posts). This distribution supports valid chi-square testing, including 2-way interactions, while maintaining adequate cell frequencies. Third, collection depth (top 10 pages on Naver, first screen on Google) reflects ecological validity, as previous behavioral studies have shown that users rarely move beyond the first page of search results [36,37].

### Data Analysis Plan

As a descriptive content analysis, the study focused on characterizing the distribution of promotional cancer information across platforms, cancer types, and institutional sources. Quantitative analysis was conducted using IBM SPSS Statistics (version 28.0) [39] for descriptive statistics and chi-square tests and Python (version 3.9) [40] for NLP-based keyword extraction.

Descriptive statistics included frequencies and percentages for all categorical variables (promotional status, intensity, institutional source, citation practices, efficacy verification, information depth, and visual usage), with extensive cross-tabulation examining relationships between variables. Chi-square tests for independence (2-tailed *α*=.05) examined associations between (1) platform and promotional prevalence; (2) cancer type and promotional rates; (3) institutional source and promotional status; and (4) promotional status and information quality indicators (citation practices, efficacy verification, information depth, and visual attribution). Fisher exact test was used when the expected cell frequencies were less than 5.

Comparative analyses identified platform-specific distributions of promotional intensity categories, cancer-specific patterns in promotional rates and institutional sources, and systematic differences in information quality characteristics between promotional and nonpromotional posts. This analytical approach reflects the descriptive and exploratory nature of content analysis research, emphasizing the identification of patterns and the examination of relationships over hypothesis testing or predictive modeling.

### Ethical Considerations

This study analyzed data obtained exclusively from publicly accessible sources and therefore did not require Institutional Review Board (IRB) approval under South Korean regulations. According to Article 16 of the Enforcement Rule of the Bioethics and Safety Act and Article 13 of its Enforcement Decree, research using publicly available materials is exempt from IRB review provided that no sensitive personal information, as defined by the Personal Information Protection Act, is collected or recorded. As this study involved no collection of personal or identifiable data, it was conducted in compliance with all applicable ethical and legal requirements.

## Results

### Sample Characteristics

The final analytical sample comprised 919 unique posts from 2 search engines. Platform distribution was as follows: Naver, 598 posts (65.1%), and Google, 321 posts (34.9%). Cancer type distribution ranged from 108 to 151 posts per type, with relatively balanced representation: prostate cancer 151 posts (16.4%), pancreatic cancer 142 posts (15.5%), colorectal cancer 134 posts (14.6%), gastric cancer 134 posts (14.6%), lung cancer 129 posts (14.0%), breast cancer 121 posts (13.2%), and liver cancer 108 posts (11.8%).

Content channels differed substantially between platforms. Naver-sourced posts were predominantly blogs (262/273, 96.0%) and café posts (11/273, 4.0%), with no hospital website content. Google-sourced posts were primarily hospital/institutional websites (141/174, 81.0%), news articles (23/174, 13.2%), and blogs (8/174, 4.6%). Overall, blogs comprised 270 posts (29.4%), hospital/institutional websites had 141 posts (15.3%), news articles had 23 posts (2.5%), and other sources had 13 posts (1.4%).

### Prevalence of Covert Promotional Content

Regarding RQ1, of the 919 posts analyzed, 447 (48.6%) contained covert promotional content (Table 3), indicating that nearly half of the analyzed cancer-related search results across platforms contained promotional elements. Platform-level comparison revealed a statistically significant difference in promotional prevalence, with Google exhibiting a higher proportion of covert promotional content (174/321, 54.2%) than Naver (273/598, 45.7%; *χ*²_1_=5.78; *P*=.02).

**Table 3.** Distribution of promotional status, intensity, and content source type by platform. For promotional intensity (n=447), *χ*²_3_=155.88; *P*<.001; Cramér V=.59, indicating a large effect.

Category/platform		Naver^a^, n (%)	Google, n (%)	Total, n (%)	Chi-square (*df*)	*P* value
Advertising status					5.78 (1)	.02
Advertising		273 (45.70)	174 (54.20)	447 (48.60)		
Nonadvertising		325 (54.30)	147 (45.80)	472 (51.40)		
Promotional intensity					155.88 (3)	<.001
Simple identification		24 (5.37)	52 (11.63)	76 (17.00)		
Partial promotion		92 (20.58)	30 (6.71)	122 (27.29)		
Full promotion		126 (28.19)	8 (1.79)	134 (29.98)		
Subtotal (advertising identified)		242 (54.14)	90 (20.13)	332 (74.27)		
Unidentified^b^		31 (6.94)	84 (18.79)	115 (25.73)		
Content source type					414.35 (4)	<.001
Blog		262 (96.00)	8 (4.60)	270 (60.40)		
Online community		11 (4.00)	0 (0.00)	11 (2.50)		
Hospital/institutional website		0 (0.00)	141 (81.00)	141 (31.50)		
News article		0 (0.00)	23 (13.20)	23 (5.10)		
Other		0 (0.00)	2 (1.10)	2 (0.40)		
Total		273 (100.00)	174 (100.00)	447 (100.00)	—^c^	—

a Naver and Google, South Korea, October 2023. N=919 content items (Naver n=598 and Google n=321). Chi-square tests of independence were conducted for each category.

b Unidentified content includes video or image-based materials, image-formatted files, and downloadable reports (eg, PDFs) requiring login or restricted access, for which text extraction and word-count analysis were not feasible. Therefore, chi-square tests excluded the ”unidentified” category due to incomparability of content format.

c Not applicable.

Among promotional posts for which character-count analysis was feasible (n=332, 74.3% of all promotional posts), promotional intensity varied substantially. Of these, 76 (22.9%) posts involved simple institutional identification, 122 (36.7%) posts contained partial promotional content (less than 50% of textual content), and 134 (40.4%) posts were dominated by full promotional messaging (50% or more of the text). Platform-specific patterns were pronounced. On Naver, promotional posts most frequently exhibited full promotion (126/242, 52.1%), followed by partial promotion (92/242, 38.0%) and simple identification (24/242, 9.9%). In contrast, Google promotional posts were most commonly characterized by simple identification (52/90, 57.8%), followed by partial promotion (30/90, 33.3%), with full promotion being relatively rare (8/90, 8.9%).

Analysis of content source types further revealed stark platform differences. Among Naver promotional posts, blogs overwhelmingly dominated (262/273, 96.0%), with the remainder consisting of online community (café) posts (11/273, 4.0%). No promotional content from hospital or institutional websites or news articles appeared in Naver search results. In contrast, Google promotional posts were primarily distributed through hospital or institutional websites (141/174, 81.0%), followed by news articles (23/174, 13.2%), blogs (8/174, 4.6%), and other sources (2/174, 1.1%). These patterns are illustrated in Figure 3.

**Figure 3.**
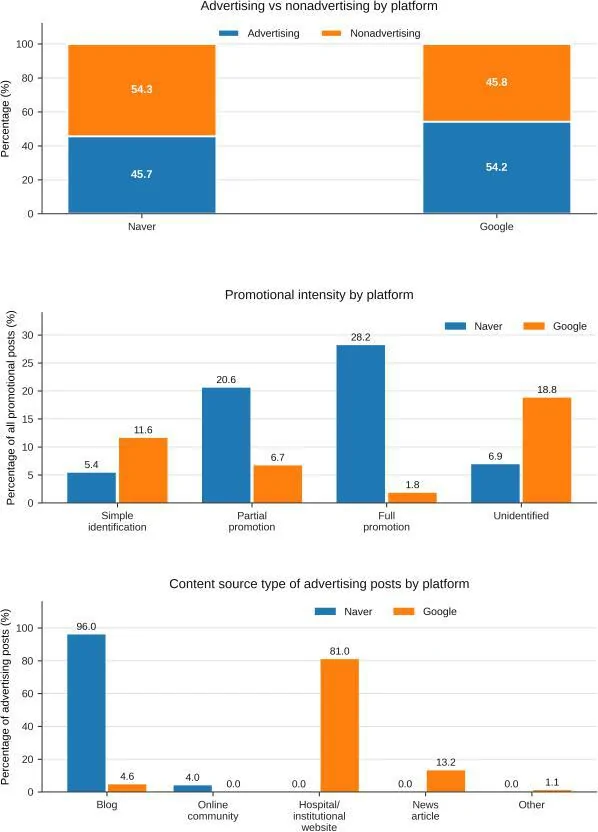
Platform differences in promotional status, intensity, and content source type. Naver and Google, South Korea, October 2023 (Content items N=919; Naver n=598 and Google n=321).

### Cancer Type

For RQ2, this study examined platform-level differences in the distribution of promotional cancer-related posts across cancer types. As shown in Table 4, substantial variation was observed in the relative concentration of promotional content between Naver and Google depending on cancer type. The strongest concentration of promotional content on Naver was observed for breast cancer, where 65 out of 79 (82.3%) promotional posts originated from Naver, compared with 14 (17.7%) posts from Google. This was followed by liver cancer, for which 27 of 36 (75.0%) promotional posts were retrieved from Naver and 9 (25.0%) posts from Google. Colorectal cancer also demonstrated a pronounced Naver dominance, with 54 of 74 (73.0%) promotional posts appearing on Naver and 20 (27.0%) posts on Google. Together, these findings indicate that promotional dissemination for breast, liver, and colorectal cancer was disproportionately concentrated within the Naver search environment.

**Table 4.** Distribution of covert promotional content by cancer type and search engine.

Cancer type	Naver^a^, n (%)	Google, n (%)	Total, n (%)
Colorectal cancer	54 (73.00)	20 (27.00)	74 (100.00)
Breast cancer^b^	65 (82.30)	14 (17.70)	79 (100.00)
Gastric cancer	39 (54.20)	33 (45.80)	72 (100.00)
Prostate cancer	32 (43.80)	41 (56.20)	73 (100.00)
Liver cancer	27 (75.00)	9 (25.00)	36 (100.00)
Lung cancer	32 (50.00)	32 (50.00)	64 (100.00)
Pancreatic cancer	24 (49.00)	25 (51.00)	49 (100.00)
Total	273 (61.10)	174 (38.90)	447 (100.00)

a Naver and Google, South Korea, October 2023. N=447 promotional content items. *χ*²₆=39.17; *P*<.001, Cramér V=.30, indicating a medium effect.

b Post hoc analyses using standardized residuals revealed that breast cancer promotional content was significantly overrepresented on Naver (z=4.2; *P*<*.*001), while prostate cancer was overrepresented on Google (z=3.3; *P*=*.*001).

In contrast, prostate cancer exhibited a higher proportion of promotional content on Google, accounting for 41 of 73 (56.2%) posts, compared with 32 (43.8%) posts on Naver. A similar pattern was observed for pancreatic cancer, where Google accounted for 25 of 49 (51.0%) promotional posts and Naver for 24 (49.0%) posts. Gastric cancer showed a more balanced distribution, with a slight predominance of promotional content retrieved through Naver (39/72, 54.2%) compared with Google (33/72, 45.8%). Lung cancer demonstrated the most even distribution of promotional content across platforms, with an identical number of posts retrieved from each search engine (32 posts each, 50.0% vs 50.0%). The complete distribution by cancer type and platform is presented in Figure 4.

**Figure 4.**
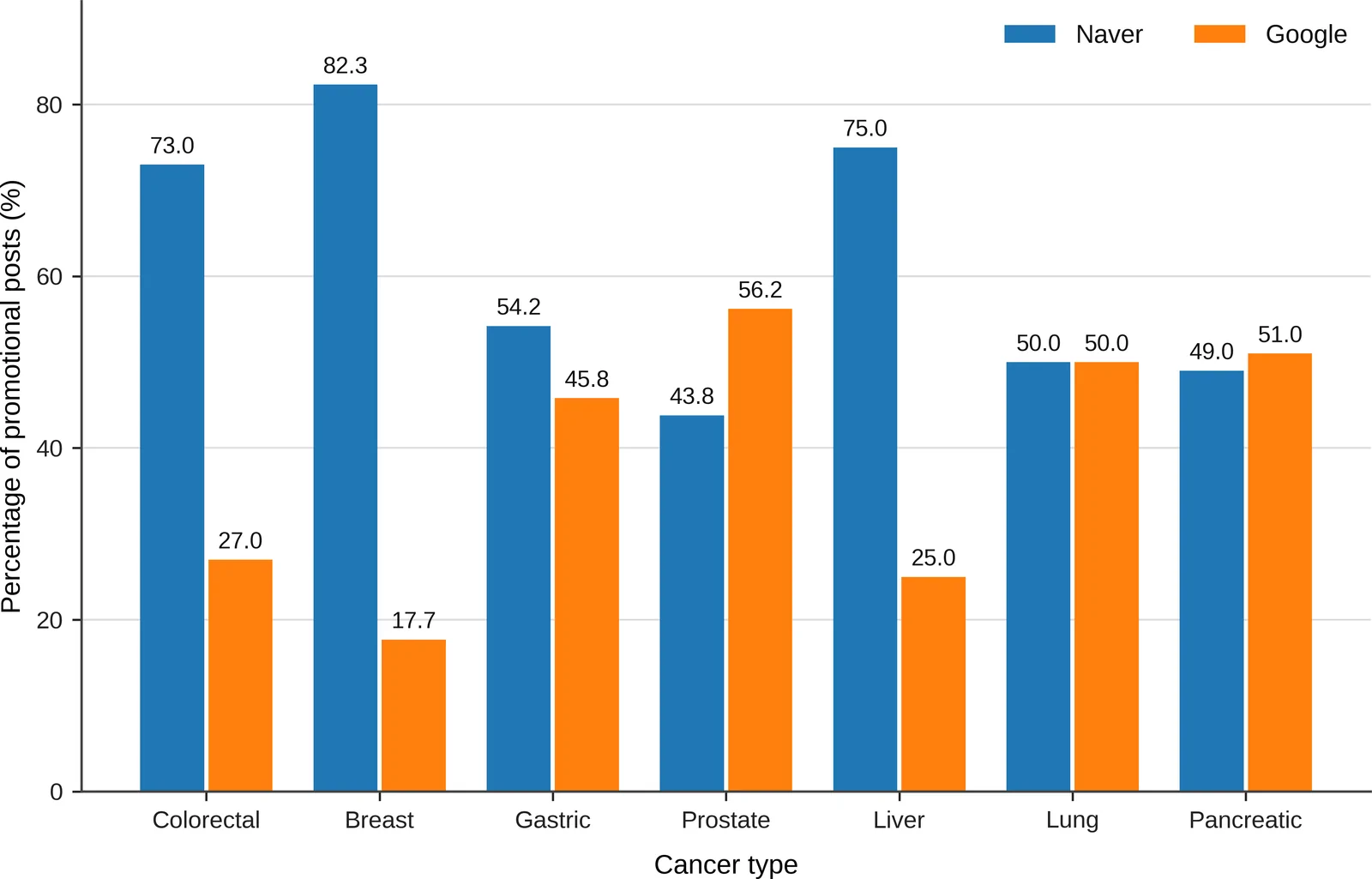
Covert promotional content by cancer type and search engine. Naver and Google, South Korea, October 2023 (promotional content items: N=447).

Further analysis by information type revealed systematic differences in how promotional content was framed across platforms (Figure 5). Across multiple cancer types, promotional content retrieved from Naver was more heavily focused on treatment- and management-related information, whereas that retrieved from Google more frequently emphasized prevention and diagnostic information. This pattern was particularly pronounced for liver and lung cancer, where Naver posts showed a strong skew toward treatment-oriented content, while Google posts allocated a greater proportion to prevention or diagnosis. In contrast, cancers such as gastric and prostate cancers exhibited more balanced distributions, though Google consistently maintained a stronger focus on diagnostic framing. These findings suggest that the platform-specific differences were observed not only in volume but also in the thematic orientation of retrieved promotional content. Overall, the association between cancer type and platform was statistically significant (*χ*²_6_=39.17; *P*<.001), underscoring substantial heterogeneity in both the prevalence and informational framing of promotional cancer-related content across search engines.

**Figure 5.**
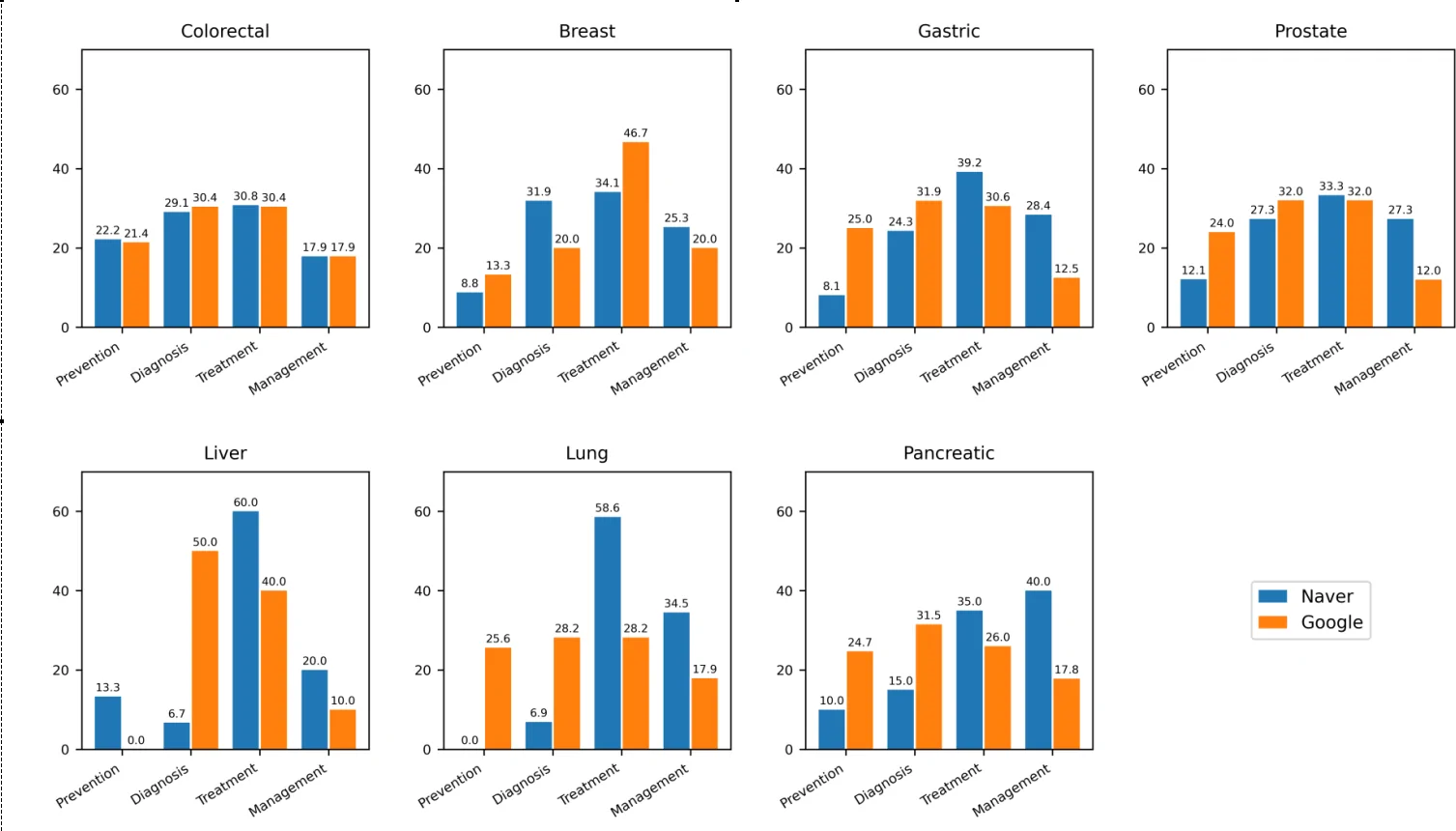
Platform differences in covert promotional content by cancer type and information category. Naver and Google, South Korea, October 2023. N=447 promotional content items.

### Institutional Source Distribution

For RQ3, the institutional origins of promotional posts differed markedly across platforms, revealing a structurally segmented pattern of medical commercialization (Table 5). Overall, traditional Korean medicine long-term care hospitals constituted the largest share of promotional content (120/447, 26.9%), followed by university-affiliated hospitals (113/447, 25.3%). Nonexperts, including patients, caregivers, and health bloggers without formal medical credentials, accounted for 95 promotional posts (21.3%), while small and medium-sized private hospitals contributed 78 posts (17.4%). Health policy and medical administration experts (27/447, 6.0%) and other sources (14/447, 3.1%) comprised comparatively smaller proportions.

**Table 5.** Institutional source distribution of covert promotional content by platform.

Type of institution	Naver^a^, n (%)	Google, n (%)	Total, n (%)
University-affiliated hospitals	17 (6.23)	96 (55.17)	113 (25.28)
Small and medium-sized private hospitals	47 (17.22)	31 (17.82)	78 (17.45)
Traditional Korean medicine long-term care hospitals	119 (43.59)	1 (0.57)	120 (26.85)
Health policy and medical administration experts	9 (3.30)	18 (10.34)	27 (6.04)
Nonexperts (general public)	78 (28.57)	17 (9.77)	95 (21.25)
Other	3 (1.10)	11 (6.32)	14 (3.13)
Total	273 (100.00)	174 (100.00)	447 (100.00)

a Naver and Google, South Korea, October 2023. N=447 promotional content items (Naver n=273, Google n=174). A *χ*² test revealed a significant association between institutional source and platform (*χ*²_5_=209.642; *P*<.001).

These aggregate distributions masked pronounced platform-specific differences. Promotional content originating from traditional Korean medicine long-term care hospitals was overwhelmingly concentrated on Naver, accounting for 119 of 120 (99.2%) posts, with only a single post retrieved from Google (0.8%). In contrast, promotional posts from university-affiliated hospitals predominantly surfaced on Google, where 96 out of 113 (85.0%) posts appeared, compared to 17 (15.0%) posts on Naver. This stark divergence indicates that distinct institutional actors occupy structurally differentiated positions within each search engine environment.

Other source categories exhibited more balanced, yet still asymmetrical, patterns. Small and medium-sized private hospitals exhibited a relatively even distribution across platforms, with Naver accounting for 47 out of 78 (60.3%) and Google for 31 out of 78 (39.7%). In contrast, nonexpert promotional posts were more frequently retrieved from Naver (78/95, 82.1%) than from Google (17/95, 17.9%). Promotional content attributed to health policy and medical administration experts appeared more often on Google (18/27, 66.7%) than on Naver (9/27, 33.3%).

These findings suggest that the dominance of traditional Korean medicine institutions and university-affiliated hospitals in promotional cancer-related content reflects not only institutional prevalence but also platform-specific structural affordances. Naver’s ecosystem appears to preferentially surface promotional content from traditional medicine providers and nonprofessional actors, whereas Google more strongly privileges promotional materials associated with large, university-affiliated hospitals and institutional expertise. This pattern underscores that institutional visibility in promotional cancer-related search results is shaped less by uniform marketing strategies and more by the infrastructural and epistemic logics embedded within each search engine.

Marked differences in the distribution of promotional sources were observed (Figure 6). Promotional content from traditional medicine institutions was almost exclusively concentrated on Naver (119/120, 99.2%), as was content produced by nonexperts (78/95, 82.1%). In contrast, promotional posts from university-affiliated hospitals were overwhelmingly retrieved from Google (96/113, 85.0%), as were posts authored by experts in health policy and medical administration (18/27, 66.7%). These findings indicate that promotional content retrieved through Naver is largely driven by noninstitutional and alternative medical actors, whereas Google preferentially surfaces promotional content originating from institutional and professional sources.

**Figure 6.**
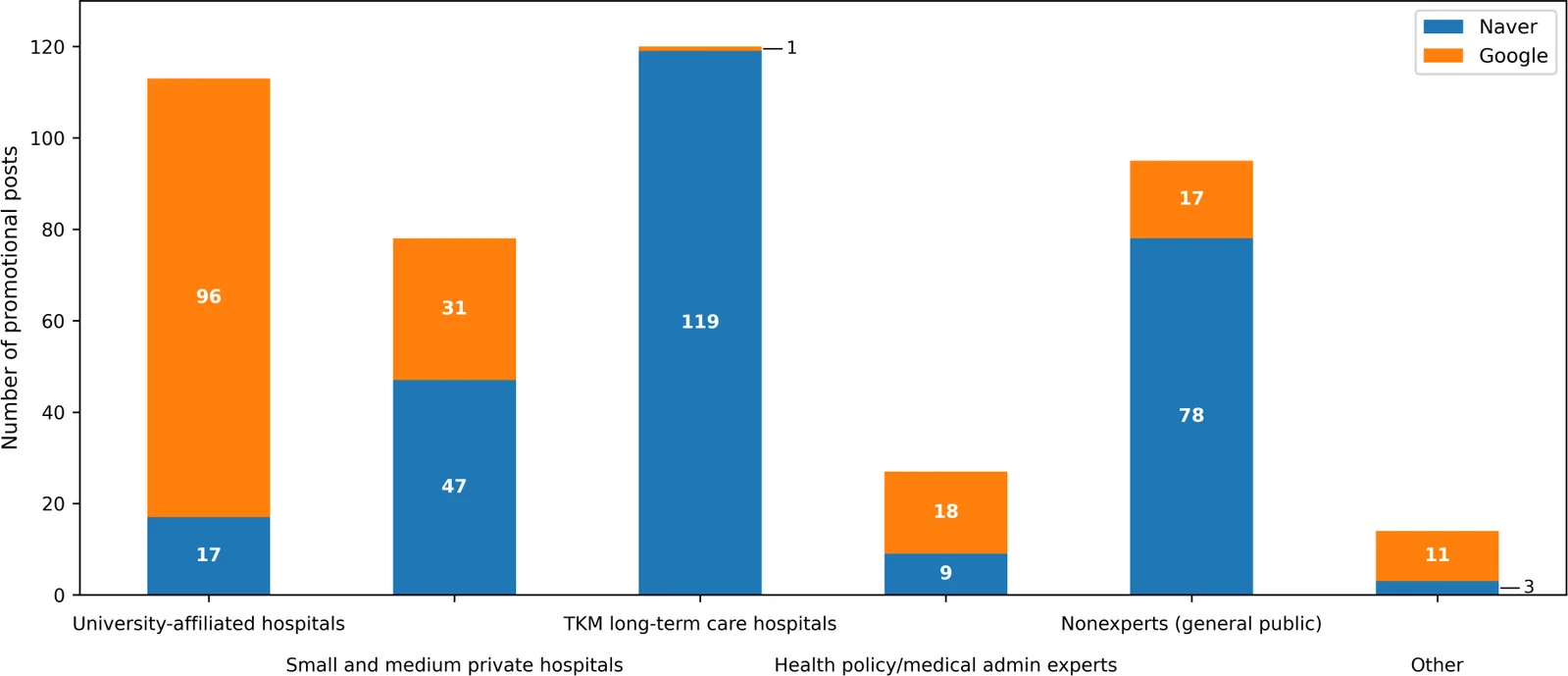
Platform differences in covert promotional content by institutional source. Naver and Google, South Korea, October 2023. N=447 promotional content items (Naver n=273 and Google n=174). TKM: traditional Korean medicine.

### Information Quality

Regarding RQ4, this study examined differences in information quality characteristics within covert, cancer-related promotional posts, focusing on citation practices, depth of information, and source attribution, and assessed how these patterns varied across different platforms. Across all promotional posts, indirect citation and limited transparency were prevalent. Overall, indirect quotation—such as paraphrased references or generalized claims without clearly identifiable sources—accounted for more than two-thirds of promotional content (302/447, 67.6%). Similarly, more than half of all promotional posts lacked explicit data source attribution (257/447, 57.5%), indicating a widespread deficit in verifiable sourcing within covert medical promotion.

Within this broader pattern of reduced informational transparency, significant platform-level differences were observed. First, citation practices revealed an overall reliance on indirect quotation across covert promotional cancer-related posts, with 67.6% (302/447) of posts using paraphrased or generalized references rather than direct quotations (Table 6). This pattern was evident on both platforms but was substantially more pronounced on Google, where indirect quotation accounted for 81.6% (142/174) of promotional posts, compared to 58.6% (160/273) on Naver. Although direct quotation was relatively more common on Naver (113/273, 41.4%) than on Google (32/174, 18.4%), these figures indicate that indirect citation constitutes the dominant referencing strategy overall. The significant association between platform and citation practice (*χ*²_1_=25.653; *P*<.001) thus reflects differences in degree rather than opposing citation norms.

Second, a similar pattern emerged for information depth. Across all promotional posts, the most prevalent format was simplified information provision without comparative evaluation, accounting for 62.6% (280/447) of the sample. This tendency was especially pronounced on Naver, where more than 3-quarters of promotional posts (210/273, 76.9%) provided only descriptive information. By contrast, Google promotional posts more frequently included comparative discussions across diagnosis, prevention, treatment, and management domains (97/174, 55.7%), although simplified formats still represented a substantial share (70/174, 40.2%). The significant chi-square result (*χ*²_2_=64.683; *P*<.001) therefore indicates not a reversal of informational norms, but a platform-specific modulation within a broader dominance of low-depth informational structures.

**Table 6.** Information quality characteristics of covert promotional content by platform.

Category		Naver^a^, n (%)	Google, n (%)	Total, n (%)	Chi-square (*df*)	*P* value
Information transparency					25.653 (1)	<.001
Direct quotation		113 (41.4)	32 (18.4)	145 (32.4)		
Indirect quotation		160 (58.6)	142 (81.6)	302 (67.6)		
Information depth					64.683 (2)	<.001
Simple information provision		210 (76.9)	70 (40.2)	280 (62.6)		
Comparative discussion of diagnosis/prevention/treatment/management		53 (19.4)	97 (55.7)	150 (33.6)		
Other		10 (3.7)	7 (4.0)	17 (3.8)		
Source attribution					86.738 (2)	<.001
Data source provided		112 (41.0)	31 (17.8)	143 (32.0)		
Data source not provided		160 (58.6)	97 (55.7)	257 (57.5)		
Not applicable		1 (0.4)	46 (26.4)	47 (10.5)		
Total		273 (100.0)	174 (100.0)	447 (100.0)	—^b^	—

a Naver and Google, South Korea, October 2023. N=447 promotional content items (Naver n=273 and Google n=174). *χ*² tests of independence were conducted for each category. Among promotional posts, 89.5% contained visual or data-based materials (eg, images, charts, or embedded datasets), while the remaining posts did not include such materials.

b Not applicable.

Third, source attribution practices demonstrated the most pronounced transparency deficit across platforms. Overall, the majority of covert promotional posts did not explicitly provide data sources (257/447, 57.5%), which is far higher than the proportion that included source attribution (143/447, 32.0%). This pattern held consistently for both Naver (160/273, 58.6%) and Google (97/174, 55.7%), indicating that the absence of explicit source disclosure is a defining characteristic of covert promotional content regardless of platform. Platform differences nonetheless emerged in format-related constraints: a substantial proportion of Google promotional posts were classified as not applicable for source attribution (46/174, 26.4%), reflecting the frequent use of nontextual or access-restricted materials such as images, videos, or downloadable reports. The association between platform and source attribution was statistically significant (*χ*²_2_=86.738; *P*<.001); however, this difference should be interpreted in the context of an overall predominance of source nondisclosure.

These results indicate that covert promotional cancer information is characterized by systematic constraints in information quality, including indirect citation, limited depth, and insufficient transparency of sources. Platform-specific affordances further shape how these limitations manifest, with Naver emphasizing overt credibility cues through quotation and Google privileging comparative framing and nontextual presentation formats that may complicate users’ ability to evaluate evidentiary reliability. These platform-specific patterns in information quality are summarized in Figure 7.

**Figure 7.**
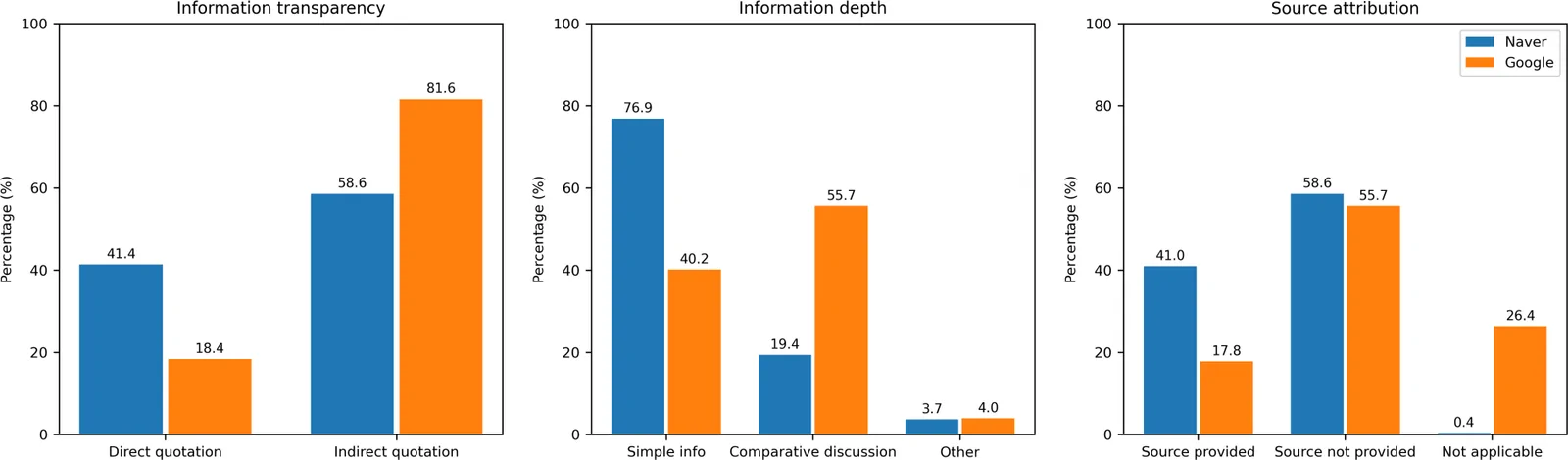
Platform differences in information quality characteristics of covert promotional content. Naver and Google, South Korea, October 2023. N=447 promotional content items (Naver n=273, Google n=174).

## Discussion

### Principal Findings

This study examined the commercialization of cancer-related information within search engine environments by comparing Naver and Google across multiple dimensions, including promotional prevalence, intensity, institutional sources, and information quality. The findings demonstrate that covert promotional content is not only pervasive but also structurally differentiated across search engines, reflecting deeper differences in platform architecture and sociocultural information practices rather than deliberate, platform-specific marketing strategies by medical institutions. These patterns should be interpreted in light of prior research on health information–seeking behavior, information quality, and native advertising in digital health environments, which emphasizes the interaction between platform design, user vulnerability, and persuasive communication.

### Structural Differentiation of Medical Commercialization Across Search Engines

A central contribution of this study lies in reconceptualizing platform differences as outcomes of structural selectivity embedded in search engine ecosystems, rather than as evidence of strategic adaptation by health care providers. Although prior research has often interpreted platform variation through the lens of organizational marketing behavior, the present findings suggest that search engines function as sociotechnical infrastructures whose structural affordances and algorithmic logics are associated with the differential visibility of particular forms of medical commercialization, not through deliberate prioritization of commercial content, but as an emergent outcome of ranking criteria that favor institutional authority, content freshness, or user engagement metrics.

Naver’s search environment, deeply integrated with blogs, online communities, and user-generated narratives, structurally favors personalized, experience-based content. Within this ecosystem, promotional cancer-related posts were more frequently authored by traditional Korean medicine institutions, private clinics, and nonprofessional individuals and tended to exhibit high promotional intensity coupled with simplified informational framing. These patterns may reflect not merely institutional choice but the structural affordances of a platform in which narrative credibility and experiential authority are culturally salient, though causal attribution to platform architecture alone requires further experimental or longitudinal evidence.

In contrast, Google’s search ecosystem—structured around institutional authority signals and domain-based ranking—tends to surface promotional content from institutional web pages with comparative informational structures. Promotional posts surfaced through Google were more often associated with tertiary hospitals, institutional websites, and news articles and exhibited lower overt promotional intensity. However, these posts frequently relied on indirect quotation practices, limited source attribution, and nontextual or access-restricted formats, such as images, videos, or downloadable reports. This suggests that Google’s algorithmic emphasis on institutional legitimacy does not necessarily translate into greater transparency of information. Moreover, institutional authority cues may create an illusion of credibility even when evidentiary clarity is limited, as reflected in the observed reliance on indirect quotation and restricted informational formats.

### Platform-Specific Manifestations of Information Quality Deficits

Across both platforms, covert promotional content exhibited systematic limitations in information quality. Indirect quotation was the dominant citation practice, explicit source attribution was frequently absent, and simplified informational formats outweighed analytically rigorous discussions. These characteristics suggest that promotional cancer-related information often falls short of standards associated with evidence-based health communication, consistent with prior assessments of online health information quality [41-43].

Within this broader pattern of information quality deficit, platform-specific differences shaped how epistemic risks were distributed. Promotional content items retrieved through Naver more frequently used direct quotations and explicit source signaling, potentially functioning as surface-level credibility cues. However, these posts predominantly provided simple descriptive information without comparative evaluation, which may limit users’ ability to assess treatment alternatives or clinical uncertainty.

Promotional content items surfaced through Google search results, by contrast, more often engaged in comparative discussions across diagnosis, prevention, treatment, and management domains. While this analytic framing may appear more informative, it was frequently accompanied by indirect citation practices and opaque source attribution, particularly in nontextual or restricted-access formats. As a result, users may encounter content that appears sophisticated yet offers limited means to verify evidentiary claims. These findings underscore that platform differences do not reflect a linear hierarchy of information quality but rather distinct configurations of epistemic vulnerability shaped by each platform’s design and governance.

### Implications for Cancer Information Seeking and User Risk

The observed patterns have significant implications for cancer information seekers, who frequently conduct online searches under conditions of emotional vulnerability, uncertainty, and time pressure. Prior research has shown that cancer-related information seeking is frequently driven by the need for reassurance and experiential understanding, leading users to rely heavily on search engines as primary gateways to medical knowledge [44-47]. Survey-based evidence further indicates that individuals with lower health literacy or limited access to professional consultation are less equipped to critically evaluate the quality of information and its persuasive intent [44,48].

Nearly 90% of promotional posts in this study included visual or data-based materials, which may serve as salience cues that amplify the persuasive impact, regardless of the evidentiary quality. This challenge persists even when disclosure elements are present. Research on social media advertising has demonstrated that consumers experience systematic difficulties in recognizing promotional content despite disclosure markers. Studies have shown no significant difference in advertising recognition between posts with explicit disclosures (eg, “sponsored” or “paid partnership with”) and those without [49]. When such materials are embedded within covert promotional formats, users may struggle to distinguish informational content from commercial persuasion, particularly when authority cues and institutional credibility markers reduce critical evaluation [50,51].

The coexistence of high promotional prevalence and limited transparency of information raises concerns about distorted expectations, selective exposure to treatment narratives, and delayed engagement with evidence-based care. These risks are amplified when platform-specific norms shape not only what information is visible but also how credibility is signaled.

### Policy Implications: the Need for Platform-Sensitive and Future-Oriented Regulation

The findings of this study indicate that prevailing approaches to regulating online medical advertising insufficiently address the structural role of search engines in shaping the commercialization of health information. Regulatory frameworks that presume a homogeneous digital environment or rely primarily on disclosure-based mechanisms fail to capture the platform-specific processes through which promotional content is produced, prioritized, and endowed with credibility [50,51].

Accordingly, effective governance of online medical promotion requires policy differentiation that is responsive to search engine architectures and sociocultural information practices. In platform environments such as Naver, where experiential narratives and informal, user-generated formats are structurally privileged, regulatory efforts should emphasize the explicit identification of promotional intent, transparency standards for testimonial-based content, and minimum evidentiary requirements for health information on blogs and community platforms. In contrast, for platforms such as Google, where institutional web pages and comparative informational structures are more prominent, policy attention should focus on strengthening source attribution standards, improving the accessibility of cited evidence, and addressing the epistemic opacity associated with nontextual or access-restricted promotional materials.

The present findings also raise broader implications for the future governance of health information in the context of generative AI. As large language models and search-integrated AI systems increasingly rely on web-based content and search engine outputs as training data and informational references, platform-specific patterns of medical commercialization may become systematically embedded within AI-mediated health information systems. Algorithmic hierarchies and sociocultural content biases associated with dominant search environments thus have the potential to be reproduced—and, in some cases, reinforced—by automated knowledge-generation processes.

Taken together, these observations underscore the need to conceptualize search engines not as neutral intermediaries, but as active infrastructures that shape both contemporary medical information markets and emerging AI-driven health communication ecosystems. Platform-sensitive regulation, coupled with forward-looking oversight of AI training data and sustained efforts to enhance digital health literacy, will be essential for mitigating the long-term risks associated with the structural commercialization of high-stakes health information [44,50,52].

### Limitations and Future Research

This study has several limitations. The analysis was based on top-ranked search results collected at a specific point in time, which may not capture temporal variation in algorithmic ranking. Additionally, nontextual promotional content classified as not applicable for source attribution could not be fully evaluated, potentially underestimating certain forms of informational opacity. Future research should incorporate longitudinal designs, user exposure measures, and experimental approaches to assess how different promotional formats influence comprehension, trust, and decision-making among patients and caregivers. Furthermore, citation practice analyses did not account for content type as a potential confounder: personal testimonials and experiential narratives may not be amenable to formal citation, and their higher prevalence on Naver could partly explain observed platform differences in citation rates. Future studies should model citation practices using statistical approaches that control for content format, thereby isolating platform-structural effects from genre-based constraints on evidentiary norms.

### Conclusion

In conclusion, this study demonstrates that the commercialization of cancer-related search results is pervasive and structurally differentiated across search engines. Differences in promotional prevalence, intensity, institutional sourcing, and information quality are shaped more by the underlying architectures and cultural logics of search platforms than by strategic marketing choices. Recognizing these structural dynamics is critical to advancing ethical health communication, protecting patients, and designing more effective, context-sensitive policies to regulate online medical advertising.

## Abbreviations

IRB: Institutional Review Board
NLP: natural language processing
RQ: research question
